# Role of Complement in Regulating Inflammation Processes in Renal and Prostate Cancers

**DOI:** 10.3390/cells10092426

**Published:** 2021-09-15

**Authors:** Giuseppe Stefano Netti, Rossana Franzin, Alessandra Stasi, Federica Spadaccino, Andrea Dello Strologo, Barbara Infante, Loreto Gesualdo, Giuseppe Castellano, Elena Ranieri, Giovanni Stallone

**Affiliations:** 1 Clinical Pathology, Center of Molecular Medicine, Department of Medical and Surgical Sciences, University of Foggia, 71122 Foggia, Italy; giuseppestefano.netti@unifg.it (G.S.N.); federica.spadaccino@unifg.it (F.S.); 2 Department of Emergency and Organ Transplantation-Nephrology, Dialysis and Transplantation Unit, University of Bari Aldo Moro, 70124 Bari, Italy; rossana.franzin@uniba.it (R.F.); alessandra.stasi@uniba.it (A.S.); loreto.gesualdo@uniba.it (L.G.); 3 Department of Medical and Surgical Sciences-Nephrology, Dialysis and Transplantation Unit, Advanced Research Center on Kidney Aging (A.R.K.A.), University of Foggia, 71122 Foggia, Italy; andrea.dellostrologo@unifg.it (A.D.S.); binfante@ospedaliriunitifoggia.it (B.I.); giuseppe.castellano@unifg.it (G.C.)

**Keywords:** complement system, cellular senescence, prostate cancer, PTX3, renal cell carcinoma, SASP, inflammaging

## Abstract

For decades, the complement system, the central pillar of innate immune response, was recognized as a protective mechanism against cancer cells and the manipulation of complement effector functions in cancer setting offered a great opportunity to improve monoclonal antibody-based cancer immunotherapies. Similarly, cellular senescence, the process of cell cycle arrest that allow DNA and tissue repair has been traditionally thought to be able to suppress tumor progression. However, in recent years, extensive research has identified the complement system and cellular senescence as two main inducers of tumour growth in the context of chronic, persistent inflammation named inflammaging. Here, we discuss the data describing the ambivalent role of senescence in cancer with a particular focus on tumors that are strongly dependent on complement activation and can be understood by a new, senescence-related point of view: prostate cancer and renal cell carcinoma.

## 1. Introduction

Recent insights into the role of inflammation in cancer contexts have completely replaced the traditional paradigms and offered opportunities for the development of new therapeutic strategies. In the tumour milieu, the complement system is released and activated both at the systemic level and locally inside parenchymal cancer cells. Furthermore, complement is defined as a double-edged sword able to promote an anti-tumour response generating an immunosuppressive microenvironment but also able to induce carcinogenesis and angiogenesis by increasing cell motility and metastasis formation. The functions of complement are diverse and can be completely opposite, depending on cancer type and stage. In addition, cancer cells have evolved mechanisms to adapt to complement surveillance (i.e., by overexpression of negative regulators as CD55, CD59, CD46) or to manipulate complement response for their advantage (i.e., by removal of C5b-9 from their membranes or by the establishment of immunosuppressive milieu by regulatory immune cells recruitment). Another central mechanism involved in the cancer context is cellular senescence, a key element of mammalian aging that can be induced by DNA damage, oxidative stress, or telomere shortening. Traditionally, senescence is considered a tumour suppressive process, both by preventing cancer cell proliferation and counteracting malignant progression. However, recent studies demonstrated that persistent senescent cells accumulation can exacerbate tumorigenesis by senescence-associated secretome that can facilitate cancer cell replication, invasiveness, angiogenesis, and epithelial-to-mesenchymal transition. Senescence could be the final event of aberrant and unbalanced complement activation.

This review attempts to highlight the complexity of complement system activation in the main settings of prostate and renal cancer and provide a comprehensive overview of the complement mediators required to support anti-cancer therapies (chemotherapy or radiotherapy). We also described the ambivalent role of cellular senescence and identified in PTX3 a possible common mediator between systemic inflammation, cancer, and premature aging. Finally, we also discussed the use of senomorphics agents (i.e., rapamycin, metformin, quercetin) that might attenuate systemic inflammation and cellular senescence in prostate and renal cancer and complement-based innovative biomarkers for early diagnosis.

## 2. Overview of the Complement System

Complement is a central part of immunity that serves as a first line of defence against pathogens and damaged host cells [1,2]. The complement system consists of more than 50 soluble and membrane-associated proteins, receptors, and regulatory factors that react in a complex network that rapidly respond to signals through a cascade of consecutive reactions triggered by the binding of pattern recognition molecules (such as C1q, mannose-binding lectin (MBL), to common bacterial moieties, specific domains on damaged cells or biomaterial surfaces [3].

Complement can be activated in the serum, in the organ parenchyma, and at intracellular level through canonical and non-canonical pathways. The final event of complement activation is the innate immune recognition by opsonization and direct lysis of pathogen, with the removal of immune complexes and apoptotic bodies, the adaptive cell stimulation and pro-inflammatory effector responses [4,5]. Complement activation can occur via three pathways: the classical, the alternative, and the lectin pathway. The classical pathway is initiated by binding of C1q to immune complexes and apoptotic cells, while the lectin is triggered by recognition of carbohydrate structures or damaged or necrotic cells. The alternative pathway is constitutively active at low levels, and acts as sentinel against pathogens or non-self surfaces that are protected by specific complement inhibitors [4,6]. Notably, the spontaneous low-level hydrolysis of C3 keeps the alternative pathway in ‘standby’ mode to allow for rapid amplification upon microbial challenge. Regardless of the initial target recognition route, a common downstream sequence of conformational changes and enzymatic reactions activation led to C3 cleavage into fragments C3a and C3b. The C3 convertase of classical and lectin pathway is indicated as C4b2b, whereas the alternative pathway C3 convertase is composed of the activation fragments of C3 and factor B (FB) (C3bBb). The downstream component C5 is cleaved by C5 convertase, thus triggering the terminal pathway, releasing C5a and leading to the terminal complement complex C5b-9 formation, known as the membrane attack complex (MAC). At the final step of the cascade, the formation of the pore-like MAC leads to osmotic cell lysis.

C3a and C5a, which are among the cleavage products of C3 and C5, act as anaphylotoxins. Anaphylatoxins are potent chemoattractans and inflammatory mediators able to promote neutrophil chemotaxis to the site of inflammation [7].

In the non-canonical pathway, several proteases, such as plasmin, can cleave C5, independently of convertase formation. An intracellular cleavage of C3 by cathepsin L also occurs independently of the cascade [8,9]. Indeed, Complosome, the intracellular complement, has been investigated mainly in human CD4^+^ T cells and correlated to homeostatic cell survival.

Considering its crucial role as a potent immune system, it is not surprising that complement activation is tightly regulated by negative regulators. A great repertoire of complement components—Factor H (FH), FI, CD35, CD46, CD55, CD59, C4BP, and C1 inhibitor (C1INH)—can prevent damage to healthy-self cells and inhibit aberrant inflammation. Unbalanced complement activation characterized a wide range of diseases—paroxysmal nocturnal hemoglobinemia, systemic lupus erythematosus (SLE), autoimmune arthritis, sepsis, Multiple sclerosis, or Parkinson disease. In the cancer context, complement has long been assumed to attack tumour cells mainly via its lytic activity. However, from recent data, complement has emerged able to promote tumor progression by upregulation of complement regulators or inducing an immunosuppressive state by recruitment of Treg and immature myeloid cells [5,10,11]. After their generation, C3a and C5a interact with their respective receptors, C3a receptor (C3aR) and C5aR1 or C5aR2 that are expressed by an extensive heterogeneity of cells, from leucocytes, to endothelial, epithelial cells or fibroblasts.

On macrophages, complement C5a increased the release of reactive oxygen species (ROS), and inflammatory cytokines (TNFα; IL-6). The complement-mediated macrophages activation plays a central role in pathogens eradication. Nevertheless, complement is also able to control proliferation, differentiation, and cellular viability for instance by C3aR and C5aR signalling pathway on antigen-presenting cells and T lymphocytes.

During tumor progression, complement anaphylatoxins are continually generated. In mouse models, C5a has been demonstrated to be a key inducer of chronic inflammation that fuels carcinogenesis. As the healthy counterpart of nucleated host cells, cancer cells often resist lytic killing by MAC. Several strategies can be adopted by cancer cells to avoid MAC-mediated lysis such as upregulating regulatory proteins, modulating the level of C5b-9 complexes on their membrane surfaces, or promoting the removal of C5b-9 [12] Several pathway are involved in C5b-9 removal from cell membranes such as protein kinases PKC and ERK, caveolin and dynamin, Hsp proteins, and the mitochondrial stress protein mortalin [13,14]. The elimination or modulation of C5b-9 from cell membranes can strongly influence cancer parenchymal cells survival and the activation of the whole innate immune system. Thereby, the understanding of the signalling that confers cell death resistance could represent a promising strategy to restore cancer cell susceptibility to attack from the complement system [5,11,15].

C5b-9 elimination and inhibition of cell death signals are mediated by caveolin and dynamin, by Hsp70 and Hsp90, by the mitochondrial stress protein mortalin, and by the protein kinases PKC and ERK. It is conceivable that various cancers and cancers at different stages of development will utilize distinct patterns of these and other MAC resistance strategies. Considering the effects of complement on cell activation and survival as well as on the modulation of the entire immune system, it is not surprising that tumours have evolved mechanisms to adapt to its presence and subvert it for their benefit.

## 3. Cellular Senescence and Cancer: Ambivalence between Anti-Tumour and Pro-Tumour Response

More than 50 years ago, Hayflick and Moorhead demonstrated the limited proliferative capacity of primary cells in culture and described as ‘replicative senescence’ the observed irreversible-age related growth arrest [12,13,16,17,18]. Years later, another type of senescence characterized by absence of proliferation, apoptosis inhibition, and the acquirement of a typical senescence-associated secretory phenotype (SASP) was discovered and identified as stress-induced senescence [19]. SASP represents the increased expression and secretion of inflammatory cytokines, chemokines, growth factors, and proteases that is crucial to maintain a senescent milieu [20,21,22]. The SASP is enriched in proinflammatory factors, such as IL-6, IL-8, CXCL1, CCL2, CCL5, and matrix metalloproteinase MMP 3 [23,24]. Regardless of age, this premature senescence can be induced by several injuries, such as activated oncogenes, radiation, genotoxic drugs, cytokines, reactive oxygen species, metabolic disturbances, and DNA damage [23,25]. In particular, DNA damage response (DDR) represents a conserved cellular mechanism of cell cycle arrest. By inducing the activation of ATM, ATR, CHK1, and CHK2 kinases, DDR response culminates in p53 stabilization, thereby in cell cycle temporary arrest, allowing DNA damage repair or in case of irreversible injury senescence or apoptosis [20,24,25]. The cell cycle arrest is established and maintained by the p53/p21 and p16INK4a/pRB tumour-suppressor pathways, which is essential to replace damaged cells that naturally accumulate over time [26,27]. For decades, this intrinsic feature of senescent cells has led to the paradigm that the senescence response is a beneficial tumour-suppressive mechanism that blocks the proliferation of pre-malignant cells at a young age [22,28,29,30]. Strikingly, a large armamentarium of senescence-inducing drugs is currently under investigation as an important anti-cancer strategy [23,31].

In a pre-clinical and clinical model of tumours, the depletion of senescence response increased susceptibility to cancer progression, and the number of senescent cells decreased from pre-malignant towards malignant lesions [23,29]. This phenomena is evident in malignant melanoma, where senescent melanocytes, despite the presence of activating mutations of BRAF^v600E^, can be silent and quiescent for years, leading to the long-term persistence of nevi [16,18,32]. Only after the loss of PTEN and/or p16INK4a, thereby after senescence-escape, the melanoma progression takes place.

The induction of a senescent state is linked to the establishment of an immunosuppressive response able to halt tumor progression or promote cancer regression. In prostate cancer, the tumor-suppressor promyelocytic leukaemia protein (PML), an essential regulator of cellular senescence, acts as a tumor suppressor and is often lost, leading to metastasis. A recent study that investigated an approach to avoid PML loss demonstrated that restoration of PML attenuated tumor growth in both prostate cancer cell lines and xenografts [18,33].

Thus, the bright side of senescent cells includes beneficial roles in promoting an immunosuppressive, anti-tumor response, recruiting immune cells and promoting tissue repair [24].

In the last decades, with ongoing new research in the field on senescence and cancer, “a dark side” with persistent accumulation of senescent cells emerged over time, promoting tumorigenesis. The consolidation of the theory of a pro-tumorigenic role of senescent cells came from evidence showing that senescent cells’ fibroblasts stimulate premalignant and malignant, but not normal, epithelial cells to proliferate in culture and accelerate tumors formation when injected in mice [34,35]. Senescent cells can exacerbate tumorigenesis by SASP or senescence-messaging secretome that is able to favour cancer cell proliferation, invasiveness, angiogenesis, and epithelial–mesenchymal transition (EMT) [23]. This is particularly evident in combination with chemotherapy. Prostate cancer patients treated with the chemotherapeutic mitoxantrone showed an increased expression of various senescence-associated genes (p16, p21) and numerous SASP markers, including IL-6 and IL-8 [36]. For that reason, the use of adjuvant senostatic interventions such as rapamycin that are not able to kill directly senescent cells but can inhibit paracrine signalling and SASP revealed therapeutic potential [37]. In a mouse model of prostate cancer treated with the chemotherapeutic mitoxantrone, rapamycin dramatically suppressed the ability of senescent fibroblasts to stimulate tumor growth in mice. More importantly, besides its role in reducing IL-6, the most relevant SASP cytokine, rapamycin was shown to also suppress translation of the membrane-bound cytokine IL1A, that allow NF-κB transcriptional activity, the central control room of all SASP cytokines [23,38,39,40].

The studies discussed reveal that cellular senescence plays an ambivalent role in cancer setting with context-dependent effects. From the initial hypothesis of senescence as a immunosuppressive agent, unbalanced SASP under persistent inflammation often in combination with chemotherapy and radiotherapy has been demonstrated to fuel SASP, leading to tumorigenesis. The use of senomorphic agents (i.e., rapamycin, metformin, quercetin) might ameliorate age-related pathologies, including late-life cancer.

## 4. Complement in Prostate Cancer

The role of the complement system in tumorigenesis is particularly relevant in the context of prostate cancer. Prostate cancer is the most common malignancy of the urinary system among men in European countries and the fifth-leading cause of death worldwide [41]. Standard treatments include surgical resection, together with radiotherapy and hormone therapy, even if these approaches are effective for early and localized prostate cancer. Incidence and mortality rates are strongly related to age with the highest incidence being seen in elderly men (>65 years of age) [42].

Based on these ethnic and aging differences, it is clear that environment factors such as diet, physical activity, and chronic inflammatory state, emerged as the central trigger in the acquired form of prostate cancers, whereas gene mutations are involved in the inherited disease [43]. Therefore, the emerging correlation between complement activation and prostate cancer development or progression is not surprising.

Supporting the link between complement activation, chronic inflammation, and prostate tumorigenesis, a large plethora of observational studies detected increased level of C3 fragments in the serum of prostate cancer patients, particularly in the elderly [44,45]. Prostate cancers are detected on the basis of elevated plasmatic levels of prostate-specific antigen (PSA > 4 ng/mL), a serine protease expressed solely in prostate tissue that acts in cancer pathophysiology by cleaving several growth regulatory proteins [44,46,47].

The ambivalent role of the complement system in promoting and counteracting tumorigenesis is particularly evident in prostate cancer [48], with a fine balance between prostate tissue and fluid prostate tissue recognized as a pro-inflammatory milieu given the function of “guardian” of the genitourinary tract able to prevent infections. In accordance, the prostate tissue of the elderly is characterized by a state of permanent, low-grade inflammation that can result in prostate tumour occurrence and progression. In contrast, prostatic fluid is anti-inflammatory because it is meant to eliminate bacteria and viruses that enter the genitourinary tract through the urethra and protect sperm from immune destruction within the vaginal tract. This is supported by evidence that no inflammatory cells can be detected in prostatic secretions of patients affected by prostatitis. In this regard, seminal plasma actually has a strong anti-complement activity.

In a pivotal study, Manning et al. used mass spectrometry to analyse the prostatic fluid obtained after prostate removal due to cancer and identify potential immunoregulatory proteins [44]. The study indicated not only the abnormal presence of C3, factor B, and clustering in prostate fluids from cancer patients but also a previously unknown C-terminal C3 cleavage product. On further analysis, the authors revealed a PSA cleavage site on C3 complement component, with a special affinity for iC3b. Intriguingly, PSA purified by its chymotrypsin-like serin protease domain was able to cleave C3 and C5 in vitro, suggesting that PSA acts as a complement activating-protease that contributes to a pro-tumorigenic environment through the chronic inflammation induced by complement activation. Moreover, the PSA mediated C3 cleaving also serves to inhibit the terminal pathway by consuming all circulating C3 [3].

Tumor cells have developed several escape mechanisms to promote tumorigenesis and metastasis, including tumor antigen downregulation, expression of molecules that inhibit T-cell viability and expansion and resistance to NK, CD8^+^T-cell granzymes, and perforin.

The overexpression of complement inhibitors and regulators is also a central mechanism to allow cancer cells survival and tumour progression [4,10,11] (Box 1). Membrane-bound Complement Regulatory Proteins (mCRPs), including a decay-accelerating factor (CD55), protectin (CD59), and membrane cofactor protein (CD46) can modulate complement activation of protective healthy self-cells from aberrant and detrimental response [49].

With regard to CD55, it is a glycosylphosphatidylinositol-linked glycoprotein able to inhibit complement lysis by accelerating the decay of C3 and C5 convertases [49].

Recently, by tissue microarray analysis in prostate tumor epithelial cells, Loberg et al. demonstrated the overexpression of CD55 in clinical specimens from patients with advanced prostate cancer, compared to a normal non-malignant prostate [50].

The emerging hypothesis was that upregulation of CD55, and subsequent inhibition of complement-mediated lysis, leads to cancer cell survival and promotes prostate cancer metastasis.

In accordance, CD55 was shown to be functionally active in human prostate cancer cell lines (PC-3) and to inhibit complement-mediated lysis in a manner dependent on its expression. To determine the role of CD55 in vivo prostate cancer tumorigenesis and metastasis, Loberg R. et al. generated PC-3 prostate cancer cells with CD55 siRNA-targeted disruption and injected these cells in a bioluminescent SCID mouse model of metastasis. The authors found that inhibition of CD55 resulted in a 76% decrease in overall tumor burden [50].

Together in the mCRP family member, CD59 inhibits the polymerization of C9 and its binding to C5b-8 through competitive inhibition of an epitope on C8, resulting in inhibition of MAC assembly and cell lysis.

Increased cytoplasmic expression of CD59 is associated with reduced survival in colorectal cancer patients [51] and with decreased overall survival in patients with adenocarcinomas of the prostate [52]. In a cohort of 86 primary adenocarcinomas, CD59 protein expression was detected in epithelia of prostate cancer, prostatic intraepithelial neoplasia, benign hyperplasia, atrophic, and normal glands. Interestingly, CD59 was strongly expressed in 36% of adenocarcinomas of the prostate and associated with disease progression and adverse patient prognosis. Similar results were obtained in another study that compared 40 biopsies of prostate cancer patients with 40 biopsies of benign prostatic hyperplasia and assessed a sharp increase in CD59 expression and colocalization with PTX3 [53].

Another highly expressed mCRP by prostate cancer cells is CD46, a transmembrane glycoprotein expressed on all nucleated cells, that functions to protect excessive complement activation by acting as a cofactor in the proteolytic cleavage of C3b and C4b, mediated by Factor I.

This tumour escape mechanism of prostate cancer cells was exploited to develop an antibody–drug conjugate able to target high positive CD46 cells and release anti-tumor agents, specifically in cancer cells [54].

In an elegant study, Su et al. demonstrated that CD46 is highly expressed in both primary and metastatic castration resistant prostate cancer specimens but not on normal tissues. Then, by sophisticated proteomic approaches, the authors investigated the internalizing pathway of anti-CD46 antibodies and identified a mechanism of tumor-selective entry via micropinocytosis, leading to outstanding selective killing of cancer cells [54].

Finally, by applying a bioinformatic tool, the ESTIMATE algorithm (Estimation of Stromal and Immune cells in Malignant Tumor tissues using Expression data) complement C7 component was shown to correlate with prognosis and identified as novel prognostic biomarkers and potential therapeutic targets for immunotherapy and small molecule drugs for prostate cancer treatment [55].

## 5. PTX3 as a Possible Link between Inflammation, Cancer, and Aging

The humoral arm of innate immunity is formed by diverse molecules, including complement components, collectins, ficolins, and pentraxins, a superfamily of evolutionary conserved proteins [56,57]. The long pentraxin PTX3 plays a pivotal role in vascular biology [58,59,60]. PTX3 acts as a functional ancestor of antibodies; it interacts with selected microbial molecules, has opsonic activity via Fc receptor, activates and regulates the complement cascade, and regulates inflammation by interacting with P-selectin via its glycosidic moiety [57,61,62]. Particularly, PTX3 interacts with complement at multiple levels. It binds C1q and activates or regulates the classic pathway of complement activation [57,60,63]. By interacting with M-ficolins and MBL, it can independently modulate the lectin pathway [64]. Regarding an alternative pathway, PTX3 has been demonstrated to bind Factor H occupying different domains not interested in complement C3 negative regulation [65].

Moreover, PTX3 promotes cellular proliferation, confers resistance to apoptosis by altering cell cycle signalling, thereby promoting cancer cell invasion and migration, and tumour escape from immunosurveillance [57,66,67,68]. It facilitates dysregulation of mitogenic signalling pathways, sustains cellular proliferation, angiogenesis, insensitivity to apoptosis, cancer cell invasion and migration, and tumor escape from immunosurveillance. Recent findings suggest that PTX3 might trigger complement enzymatic cascade in a self-feeding cycle of chronic inflammation and tumorigenesis.

A large body of evidence demonstrated the increased tissue and serum expression of PTX3 in prostate cancer patients, compared to prostatic inflammation and benign prostatic hyperplasia (BPH), thereby encouraging PTX3 evaluation in clinical setting as a central biomarker able to discriminate between the different features of prostate diseases and to predict cancer development [66,67].

Besides the role of PTX3 as a biomarker, recent studies have demonstrated that PTX3 increased expression in prostate cancer biopsies colocalized with C1q deposits and correlated with augmented C3aR and C5aR receptors. The role of C5aR receptors in the promotion of cellular survival and apoptosis resistance has been extensively investigated in the presence of PTX3. This alteration might lead to the PTX3-mediated promotion of cellular proliferation, angiogenesis, and insensitivity to apoptosis, possibly leading to cancer cell invasion and migration [53]. In accordance with the concept of complement as an enhancer of tumour promotion, no colocalization between PTX3 and terminal C5b-9 was assessed, indicating the possible elimination of C5b-9 from the cancer cell surface or interference with C5b-9 stability to maintain carcinogenesis.

The observed increased tissue and serum level of PTX3 could also be interpreted as an attempt of the innate immune system to induce a complex and coordinated inflammatory/reparative response [69]. It has recently emerged that the increase in complement components’ concentration does not automatically assign a pro-tumorigenic role to the complement system. It can thus be reasoned that a high level of complement components could be associated with an augmented number of target cells and the inflammatory milieu in the tumor microenvironment could be kept by downregulation of complement inhibitors, specifically Factor H and Factor I, especially under hypoxic conditions.

Indeed, in an elegant study, Bonavita et al. demonstrated that PTX3 act as an extrinsic oncosuppressor gene able to block complement-induced tumorigenesis [70,71]. In PTX3-deficient mice, the authors showed an increased susceptibility to mesenchymal and epithelial carcinogenesis, complement C3 deposition and C5a circulating levels, macrophage infiltration, cytokine production, and angiogenesis. These results support the theory that PTX3 can control C3-dependent complement activation and modulate cancer-related inflammation [71]. Interestingly, macrophage accumulation and cytokine production were completely reversed in C3-deficient or C3/PTX3-double deficient mice [71]. Intriguingly, PTX3-deficient mice were characterized by important genetic instability, in particular displaying a high frequency of p53 mutations, oxidative DNA damage, and expression of DNA damage response (DDR) markers. These findings are particularly relevant, considering that DNA damage is a key event that leads to cancer initiation and progression and DDR response represents an essential defence to prevent malignant transformation [18,22,72,73,74]. The DDR activation can ultimately lead to cellular senescence, or if the genetic mutations are not repaired to cancer progression and represent a crucial bifurcation point in the destiny of the cells at the interface between aging and cancer (Box 1).

Another layer of complexity is added by the fact that PTX3 expression is epigenetically regulated in select human tumors (e.g., leiomyosarcomas and colorectal cancer) by methylation of the promoter region, a process that could be also controlled by an aged microenvironment [75]. Importantly, these findings can correlate the complement activation with the establishment of a senescent, pro-aging environment characterized by apoptosis inhibition and the acquirement of a specific secretory phenotype called SASP in a chronic, persistent low grade inflammatory state defined as inflammaging.

In accordance with these observations, aging was associated with an important alterations in the systemic inflammatory milieu, in particular with decreased plasma PTX3 (*p* ≤ 0.050) [76].

In other age-related contexts, PTX3 seems to play a pivotal role in the deposition and remodelling of bone matrix during the mineralization process, promoting osteoblasts differentiation and activity, suggesting a pivotal role in the pathophysiology of age-related bone diseases, such as osteoporosis, both in mice and humans [77].

Furthermore, given its involvement in bone metabolism, several studies agree with the definition of PTX3 as a molecule significantly involved in the pathogenesis of age-related bone diseases, such as osteoporosis, both in mice and humans.

Finally, a protective role for PTX3 in response to complement dysregulation was also demonstrated in age-related macular degeneration (AMD), the leading cause of blindness worldwide. Here, PTX3 can form a ternary complex with C3b and Factor H, inhibiting an alternative pathway, thereby reducing inflammation at the level of retinal pigment epithelium [78].

In conclusion, in prostate cancers, elevated PTX3 levels could reflect the attempt of a reparative process aimed to counteract systemic inflammation or genetic instability that drives carcinogenesis.

In preclinical models, PTX3 functions as an extrinsic oncosuppressor gene, reducing complement-driven macrophage-mediated tumour promotion and suggesting the potential role as an immunosuppressive agent through the establishment of cellular senescence.

## 6. Complement in Renal Cell Carcinoma

Renal cell cancer (RCC) is the cause of over 140,000 deaths per year [79]. RCC includes different histologic subtypes, with clear-cell RCC (ccRCC) accounting for around 85% of all the cases [80,81]. Despite localized ccRCC being treated by partial or total surgical ablation of the kidney, metastatic ccRCC remains a clinical challenge, with 5-year overall survival rates of 0–20%. In addition to high vascularization (due, in part, to the Von Hippel-Lindau, VHL mutations), many ccRCC tumors have an important inflammatory cell infiltrate [82,83,84,85,86,87,88].

We have already discussed the role of the complement system in promoting cancer progression by activating angiogenesis and driving immunosuppression. It should be noted that this capacity is further amplified in renal carcinoma, since kidney glomerular and tubular cells can virtually produce all complement components [82,83,89,90,91,92,93].

In the ccRCC, a predominant role for classical pathway activation was demonstrated in primary tumors from different patient cohorts (n = 264), ccRCC cell lines, and complement-deficient mice [3,94]. In particular, tumor cells were shown to produce C1r, C1s, C4, and C3, whereas C1r and C1s were able to take over the tumor-associated macrophages-produced C1q leading to formation of the initiating C1 complex (Box 1). Strikingly, mice deficient in C1q, C4, or C3 displayed decreased tumour growth. Furthermore, the ccRCC tumors infiltrated with high densities of C1q-producing tumor-associated macrophages revealed an immunosuppressed microenvironment, characterized by high expression of immune checkpoints (i.e., PD-1, Lag-3, PD-L1, and PD-L2), and associated poor clinical outcome.

More recently, Daugan MV et al. highlighted the prognostic value of C1s and C4d in renal cell carcinoma, assessing the dual role of C1s in promoting ccRCC progression and in modulating cancer phenotype in a complement cascade-independent, noncanonical manner [95,96]. Similar results were obtained for Factor H, when activated intracellularly, it promoted cancer cell survival and migration [97]. These results confirmed the pivotal role of complement to drive renal cell carcinoma progression and influence tumor microenvironment towards an immunosuppressive state. Furthermore, these studies shed new light on the complexity of the complement system, that could be activated also intracellularly and exert non-canonical, non-immune related, and complement cascade independent functions [98]. The detection of complement activation and immune cell infiltrates on renal cell carcinoma primary biopsies may serve as a new predictive factor for immunotherapy [99].

Accordingly, tumor-associated macrophages displayed an anti-inflammatory M2-like functions, thus promoting cancer cell proliferation, angiogenesis, metastasis, and T-cell exhaustion. These cells were initially characterized using CD163 and CD204 as markers and their infiltration was associated with poor clinical prognosis [100]. More recently, a CD38^+^CD204^+^CD206^−^ profile of tumor-associated macrophages have been in-depth correlated with immunosuppression [101].

The complement system has been found to have a vital impact on tumor initiation and progression with C5a anaphylatoxin playing a central role. In a seminal study, Xi W et al. provided evidence on the role of C5a as an independent adverse prognostic biomarker for clinical outcomes of ccRCC patients after nephrectomy. The authors retrospectively enrolled 272 ccRCC patients undergoing nephrectomy and correlated C5a level with clinicopathologic features and prognosis. In addition, tumoral C5a could significantly stratify patients’ prognosis both in advanced stage (TNM III + IV) and intermediate/high risk group (SSIGN score ≥ 4) [102]. In order to provide a potential molecular mechanism, the authors investigated the C5a-C5aR axis, one of the key pathways in regulating malignancy.

It should be considered that C5aR is ubiquitously expressed, especially in cancer cells, and represents an often-underestimated mechanism of direct interaction of C5a with C5aR on cancer cells. More importantly, C5aR contributes to metastasis by suppressing effector CD8^+^ and CD4^+^T-cell responses in breast cancer and lungs. Mechanisms of this C5aR-mediated immunosuppression suppression involved recruitment of immature myeloid cells (MDSCs), generation of T_reg_ cells, and regulation of TGFβ and IL10 production [103,104]. Notably, C5a also functions as a boosting factor in MDSCs’ important immunosuppressive cells that restrain T-cell proliferation and function. Importantly, pharmacologic blockade of C5aR or its genetic ablation in C5aR-deficient mice were sufficient to reduce lung metastases [103,105,106,107,108].

Another complement component observed highly expressed in serum and tissue level in ccRCC patients is the already-discussed PTX3. In a 10-year retrospective cohort of ccRCC patients, PTX3 was demonstrated to co-localize with C1q, C3aR, C5R1, and CD59, but not with C5b-9 terminal complex. In addition, patients with higher PTX3 levels also showed significantly lower survival rates. These results indicated that PTX3 plays a key role in the promotion of an pro-inflammatory/pro-carcinogenic state in the ccRCC microenvironment, by activating the classical pathway of the complement system [109].

In renal clear cell carcinoma patients, looking into mCRP, Blok V et al. determined the cellular localization and level of CD46, CD55, and CD59. The augmented expression of CD55 and CD59 suggested a predominant role in progression of ccRCC, whereas the amount of CD46 was significantly associated to tumour stage. The histological findings of C3d and C5b-9 on renal tumor cells led to the hypothesis that the complement system could be involved in the immune response against ccRCC [107] expression level and cellular distribution of CD46, CD55, and CD59 on renal clear cell carcinoma cells. The authors found that expression of CD55 and CD59 is increased in renal clear cell carcinoma, which may contribute to the progression of these tumors. Particularly, the expression level of CD46 was found to be significantly associated with tumor stage. Furthermore, frequently C3d and occasionally C5b-9 were present on renal tumor cells in situ, suggesting that the complement system is involved in the immune reaction against RCC. A statistically significant association was observed between a high C3d deposition and a low expression level of CD46. Based on these findings, the blocking of mCRP could represent a promising strategy to enhance the efficacy of potential mAb-mediated immunotherapy of RCC [110].

Box 1 **Role of the complement system in promoting tumorigenesis in prostate and renal cancer.**

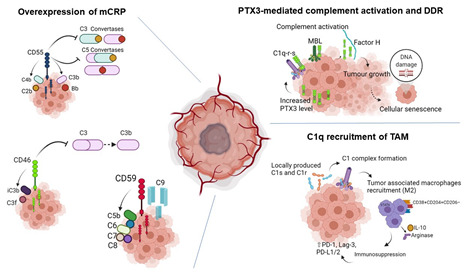

**Overexpression of mCRP:** mCRPs such as CD55, CD46, and CD59 exert protective, regulatory functions on complement system activation and can prevent damage to healthy-self cells, thus inhibiting aberrant inflammation. The overexpression of complement inhibitors and regulators is a central mechanism to allow cancer cells’ survival and tumor progression.

The CD55 (also known as DAF, decay-accelerating factor) glycoprotein recognizes C4b and C3b fragments that are generated during activation of C4 (classical or lectin pathway) or C3 (alternative pathway). Interaction of CD55 with cell-associated C4b of the classical and lectin pathways prevents formation of the C4b2b C3-convertase (blue and yellow, upper part right), whereas interaction of DAF with C3b of the alternative pathway interferes with the conversion of factor B to Bb by factor D, thereby preventing formation of the C3bBb C3 convertase of the alternative pathway (C3 convertase purple and red, high left). Thus, by limiting the amplification convertases of the complement cascade, DAF indirectly blocks the formation of C5 convertase, subsequently of MAC. Overexpression of CD55 has been assessed in clinical specimens from patients with advanced prostate cancer, compared to normal non-malignant prostate and in clear cell renal carcinoma.

CD46 (also known as membrane cofactor protein) is a transmembrane glycoprotein that acts as a cofactor in the proteolytic cleavage of C3b and C4b. CD46 is highly expressed in both primary and metastatic castration-resistant prostate cancer specimens and was associated to tumor stage in renal clear cell carcinoma.

CD59 (also known as protectin) can prevent C9 from polymerizing and forming the complement membrane attack complex. CD59 tumor overexpression is correlated with decreased overall survival in patients with adenocarcinomas of the prostate.

**PTX3 mediated-complement activation:** PTX3 can activate all three complement pathways. PTX3 binds C1q and activates or regulates the classic pathway, by interacting with M-ficolins and MBL can modulate the lectin pathway; moreover, at a soluble level, PTX3 interacts with Factor H. In prostate cancer and renal cell carcinoma, increased tissue and serum expression of PTX3 has been assessed and correlated with poor prognosis. However, from another perspective, PTX3 can act as an extrinsic oncosuppressor gene able to modulate complement-induced tumorigenesis and DNA damage associated with genetic instability of the tumor microenvironment.

**C1q mediated recruitment of TAM:** In renal cell carcinoma, tumor cells were shown to produce high levels of C1r, C1s, C4, and C3. The C1r and C1s together with C1q released by local TAM led to the formation of C1 complex. The more C1 complex was formed, more of TAM infiltrated the renal carcinoma tissue. In ccRCC, high densities of C1q-producing TAM revealed an immunosuppressed microenvironment, characterized by high expression of immune checkpoints (i.e., PD-1, Lag-3, PD-L1, and PD-L2), and associated with poor clinical outcomes.

Tumor-associated macrophages displayed anti-inflammatory M2-like functions. These cells were initially characterized using CD163 and CD204 as markers and their infiltration was associated with poor clinical prognosis. More recently, a CD38^+^CD204^+^CD206^−^ profile of TAM was in-depth correlated with immunosuppression.

**Abbreviations:** TAM: Tumor-associated Macrophages, mCRP: Membrane-bound Complement Regulatory Proteins, DDR: DNA damage response

## 7. Complement Potentiates Cancer Therapy, including Antibody-Mediated Cytotoxicity, Vaccines, and Radiotherapy

In the tumor field, the complement system is defined as a double-edged sword: on one side promoting cancer development, while on the other enhancing anti-tumor immune response. Several studies reported that the local synthesis and activation of complement cascade has a central role in tumoral immune response, compared to liver-derived complement components, underling that cancer cells are primarily involved in complement activation and tumor biology [3]. In particular, the expression of the anaphylatoxin receptors C3aR and C5aR1 increased tumor progression and thus they could be targeted for anti-tumorigenic therapy [5]. Experimental studies showed that the use of C5aR1 inhibitors led to reduced tumor growth in different tumor mouse models [5]. Accordingly, in vitro data demonstrated the efficacy of these inhibitors in reducing activation of the C5a–C5aR1 axis in human cancer cells, decreasing their viability. Recently, an ongoing clinical trial of phase I, STELLAR-001, NCT03665129, is now recruiting patients with advanced solid tumors to treat them with C5aR1 monoclonal antibody (IPH5401) in combination with the immune checkpoint inhibitor, anti-programmed cell death 1 (PD1) [111]. Moreover, therapeutic inhibition of C5aR with the small peptide antagonist PMX-53 has been found to increase the effects of paclitaxel chemotherapy, enhancing T cell anti-tumoral response in neoplastic tissue [104]. Interestingly, the use of C3 inhibitor abrogated the immunosuppressive response of intra-tumor neutrophils, suggesting that complement inhibition could be a promising therapeutic strategy for ovarian cancer in which suppressive neutrophils impaired anti-tumor immunity response [112].

Other relevant studies indicated that complement activation is essential for a robust anti-tumor response. In particular, monoclonal antibody-based immunotherapy was considered a versatile and powerful strategic therapy to drive complement activation on targeted cancer cells mediating cell cycle arrest or apoptosis [113,114,115,116,117,118]. The chimeric anti-CD20 antibody, called Rituximab is the first one used in routine cancer therapy. Kennedy et al. demonstrated that Rituximab facilitated C3b deposition on CD20+ cells in the presence of normal human serum (NSH), promoting complement-mediated cellular lysis [113]. Moreover, Cragg et al. confirmed that the effect of Rituximab depends on complement activation and the use of cobra venom factor (CVF) impaired the therapeutic activity of this antibody in two lymphoma xenograft models [114]. Another experimental study showed that Rituximab efficacy is strongly associated with complement activation rather than macrophages, T cells, and neutrophil response in a mouse model of B-cell non-Hodgkin lymphoma [118].

Despite these relevant results, the use of monoclonal antibodies in cancer therapy shows several limitations. Cancer cells developed chemoresistance, downregulating the expression of the target epitopes, recognized by monoclonal antibodies, avoiding complement activation [119].

Another strategy to optimize anti-tumor therapy is the generation of bispecific antibodies able to recognize two different epitopes—one directed against tumor-specific antigen and the other versus regulator sites for complement activation [120]. This approach showed prominent results in the treatment of several tumors, such as colorectal cancer cells, renal cell carcinoma (RCC), and CD20 positive lymphoma [120]. In the RCC setting, Sier et al. demonstrated that the use of bispecific antibodies able to bind to G250 antigen on one side, highly expressed on clear-cell RCC, and on the other hand the CD55 molecule, which belongs to membrane-bound complement regulatory proteins (mCRP) family, improved carcinoma cell killing [118]. Thus, the following approach specifically targets RCC cells and inhibits mCRP function, avoiding the inhibition of complement cascade [119]. In contrast, Reese et al. found that complement promotes RCC growth through the inhibition of antitumor immunity by the recruitment of immunosuppressive cells within tumours [82]. In addition, a recent work demonstrated that the C5a/C5a receptor 1 (C5aR1) axis could be considered a prognostic value in human RCC [99]. Otherwise, high expression of complement genes was correlated with good prognosis in liver, breast, pancreatic, and cervical carcinomas. Thus, complement inhibition might not be a favorable strategy for all tumor types, but in general can be considered a good target for cancer therapy.

Macor et al. tested two types of bispecific antibodies, CD20/CD55 (MB20/55) and CD20/CD59 (MB20/59)—each of them composed by anti-complement regulator antibody (anti-mCRP) and anti-tumour specific antibody, for the treatment of CD20 positive lymphoma [119]. Both treatments showed beneficial effects with increased immune response against tumor cells [119].

An alternative strategy to optimize complement activation on targeted tumor cells was obtained by a new generation of monoclonal antibodies composed of anti-tumor specific antibody and complement-activating proteins such as CVF or C3b to overcome tumor resistance [120]. A combined approach with different antibodies targeting several non-competing epitopes reached significant results by augmenting the surface for classical complement activation [121]. Indeed, Mamidi et al. demonstrated that the infusion of several variants of anti-HER-2 was more effective in inducing CDC when compared to a single antibody anti-HER-2 infusion [122]. Another elegant study showed that an engineering antibody composed by CR2 (CD21) and Fc region of IgG1 binds C3d deposits on tumor cells and enhanced CDC activation via the Fc portion, and mediates the recognization and recruitment of effector immune cells to metastatic cancer cells [123]. This synergetic action enables the complement system and host immune response to counteract tumor progression. In an immunostimulatory context, these therapeutic strategies could achieve powerful anti-tumor effects and overcome cancer resistance [123]. The potential benefits are context-dependent and have to be evaluated for each subset of patients and tumor type.

In the context of these new approaches, recent studies have indicated that complement activation could increase vaccine efficacy in the cancer context [124]. As is well known, C5a was able to increase antigen expression with dendritic cells and co-stimulatory mediators [125]. Floreani et al. performed a preclinical mouse model of melanoma to evaluate the efficacy of a peptide-based cancer vaccine with C5a agonist to limit cancer progression [126]. They found that tumor growth was reduced, and survival enhanced in immunized mice [126]. These results point to another perspective in cancer therapy, considering the complement system as a good target in tumor immunotherapy, in particular for vaccine design.

Many complement factors are also involved in radiotherapy treatment to reach an efficient anti-tumor host response [127,128,129]. Emerging evidence correlates the effects of radiotherapy in inducing tumor cellular damage, increasing inflammatory milieu, and the immune response against tumor cells [127,128,129]. Indeed, radiations are able to potentiate complement activation within solid tumors and increasing infiltration of immune cells. In addition, the release of C5a and C3a modulate DC responses that subsequently activated CD8^+^ effector T cells within irradiated solid tumor [129]. All these studies support the use of radiotherapeutic approach to enforce complement activation in solid tumors, destroying malignant cells.

## 8. Complement-Based Biomarkers for Cancer Prognosis

Despite there being a strong difference in expression profile among genes encoding complement mediators, a recent in silico analysis did not show a great heterogeneity between cancer types. Complement C3 with other components of the classical pathway, such as C1QA, C1QB, C1QC, C1R, C1S, C4A, and C2 are found expressed in several cancer cell types [10]. Otherwise, genes encoding factors of lectin cascade are hardly expressed [10]. The components of alternative pathway are heterogeneously expressed, and lower levels are found in some cancer types, such as chromophobe renal cell cancer and prostate adenocarcinoma [10]. Therefore, these findings underline that complement activation in all cancer types generally occurs via classical and alternative pathways.

For several decades, complement activation has been considered fundamental for killing of malignant cells and cancer immune surveillance [130]. It is important to speculate that complement activation is involved in preventing cancer progression [130]. Indeed, abundant data in the sarcoma field has demonstrated that complement cascade is involved in the antitumoral response of B lymphocytes via its activation on deposited immune complexes [131,132]. Moreover, C5 expression was associated with overall survival in Ewing’s sarcoma [133]. Further studies are needed to clarify the correlation between complement activation and tumor progression for cancer types in which complement has been described as a protective mediator of tumor arrest.

As previously underlined, complement activation has been also associated with tumor progression and its mediators could have a prognostic impact on overall patient survival [3]. Among cancer types with a detrimental complement activation, the in-silico analysis revealed that complement genes were associated with a worse outcome [3]. In particular, Reese et al. performed the systematic analysis of expression of several complement genes in human solid tumors and found high expression of 11 complement genes in RCC patients with unfavorable prognosis [82]. Among them, complement factor B (CFB), C5AR1, CFH, C3, C1R, C1S C1QA, and C1QB were strongly upregulated in an aggressive inflammatory subtype of RCC. Conversely, complement regulatory genes (CD46, CD55, and CD59) were downregulated [82]. Recently our group demonstrated in a 10-year retrospective cohort study that PTX3 expression was elevated in both neoplastic renal cell lines and tissues and strongly associated with complement system activation and tumor progression [106]. Therefore, it is evident that the persistence of a pro-inflammatory milieu determines the basal condition for an uncontrolled RCC proliferative response.

Accordingly, we also demonstrated the role of PTX3 in prostate cancer development, highlighting the link between inflammation, complement activation, and neoplasia progression [53]. In our study, we did not observe the C5-b9 formation that is necessary for neoplastic cells lysis [53]. Nevertheless, even if the activation of complement cascade is incomplete, the release of intermediate inflammatory mediators could promote neoplastic cell survival and proliferation. Recently, Chen et al. analyzed the gene expression profile of 495 prostate cancer samples from the Cancer Genome Atlas (TCGA) database (https://genomecancer.ucsc.edu/) [55]. Interestingly, they found an increased expression of complement factor C7 in patients with improved prognosis [55]. Considering that C7 can form the terminal complex C5-b9, leading to cellular lysis, C7 could be a potential tumor suppressor and a biomarker of good prognosis [55]. In accordance, Ying et al. demonstrated that decreased levels of C7 within non-small cell lung cancer were correlated to tumor progression and worsen prognosis [134]. Despite these findings, very limited data related to complement and human prostate cancer progression are available.

In another context, Nabizadeh et al. recently showed that knockout mice for C3aR were protected from melanoma progression and presented a significant infiltration of inflammatory cells and T lymphocytes within tumor [135].

In cancer, complement mediators could be downregulated and upregulated and these modifications are not only found locally, but also in systemic compartments. Moreover, the increase or decrease of some complement factors within tumors did not obviously reflect a similar condition in systemic compartments [3]. Current evidence suggests that liver-derived complement seems to be less important in tumor biology compared to locally and/or intracellularly derived complement [3]. Further studies are needed to correlate plasmatic compartment with the tumor microenvironment in order to design efficient complement-targeted therapeutics for the appropriate type of cancer.

## 9. Conclusions

In the recent literature, the involvement of the complement system as a crucial mediator in prostate and clear cell renal carcinoma is extremely clear. Complement’s anti- or pro-tumorigenic properties are strongly dependent on the type and stage of tumor, physiological context, duration of the injury, and persistence of chronic inflammation. Complement is defined as a double-edged sword able to promote an anti-tumour response generating an immunosuppressive microenvironment, but also able to induce carcinogenesis and angiogenesis by increasing cell motility and metastasis formation. Furthermore, cancer cells evolved mechanisms to adapt to complement surveillance (i.e., by overexpression of negative regulators as CD55. CD59, CD46) or to manipulate complement response for their advantage (i.e., by removal of C5b-9 from their membranes or by the establishment of immunosuppressive milieu).

For instance, PTX3 increased levels in serum and biopsies of prostate and renal cancer patients reflected the attempt of a reparative process aimed to counteract systemic inflammation or the DDR-mediated genetic instability that accelerates carcinogenesis. This is line with findings in preclinical models showing that PTX3 functions as an extrinsic oncosuppressor gene, suggesting a potential role as immunosuppressive agent through the establishment of cellular senescence.

Moreover, cellular senescence also has an ambivalent role in cancer setting with context-dependent effects. When the cell cycle arrest that is associated with senescence is a key tumor-suppressing mechanism, SASP is associated with detrimental carcinogenesis. Unbalanced SASP with persistent inflammation, often in combination with complement activation after chemotherapy and radiotherapy, has been demonstrated to fuel malignant transformation. Understanding the different pathways and cytokines (i.e., IL-6, CXCL-1, PAI-1, MCP-1) that regulate SASP may enable us to identify potential targets for pharmacological inhibition. The clinical use of senomorphics agents (i.e., rapamycin, metformin, quercetin) might increase the arsenal available to treat senescence-associated and complement-related pathologies.
